# A word for the nephrologist: what we changed in our habits during the COVID-19 pandemic?

**DOI:** 10.11604/pamj.supp.2020.35.2.24202

**Published:** 2020-06-25

**Authors:** Dina ibrahim Montasser, Younes Skri, Benkirane Oussama, Carla sanama, Taoufiq aatif, Driss el kabbaj

**Affiliations:** 1 Department of nephrology of Military Hospital of Instruction Mohammed V, University of medicine and pharmacology, Rabat, Morocco

**Keywords:** COVID-19, unit of dialysis

## To the Editors of the Panafrican Medical Journal

If the WHO declared in March 2020 a state of pandemic influenza COVID-19; we were hardly prepared for such an exceptional and surealist situation, but one that required a lot of creativity and bravery from our team of nephrologists and nurses who provide dialysis for 50 patients / week. We advise the nephrological community: To remain united, organized, reassuring and reassured.

What we adopted in our context and within our means: 1) An organization that can be modified according to the national and local evolution of the epidemic. The protective barrier measures starting with the regular washing of the hands whenever possible with water and with the knowledge of the patients and the nursing staff, and the use of hydro-alcoholic gel, ensure the distance, and avoid the contacts thus that the systematic wearing of a mask for all nursing staff. 2) A 50% reduction in care capital with a well-defined and clear distribution of tasks because of the conversion of hospital activity to the care of COVID-19 patients. 3) Limitation of the movement of people in structures. 4) A regular cleaning of surfaces and generators before and after each session. 5) Prohibition of food during the sessions. 6) A systematic measurement of the temperature of each patient with validation of a rapid standardized questionnaire eliminating the daily exposure and the clinical symptoms most frequently found in the flu COVID-19 before its access to the hemodialysis center. 7) Education of the patients concerning the protective measures and the procedure started if they suspect the appearance of symptoms of the disease. 8) We did not advise stopping blockers of the renin angiotensin system [1]. 9) Dialysis is indicated for COVID-19 patients presenting acute renal failure and for chronic hemodialysis patients. 10) We reduce the number of sessions from 3 sessions to 2 sessions per week. 11) Handmade visors were manufactured and offered by our nurses and distributed for the nursing staff and for the patients. 12) A system of rotation of the nursing staff in contact with COVID-19 patient by working 15 days then confinement 15 days then realization of a PCR to be set up for all the staff before joining the non COVID-19 sector. 13) An encouragement of remote teleconsultation mainly of nephrology patients, peritoneal dialysis and transplantation with the possibility of delivery of medicines to transplant patients´ homes. 14) A collaboration with doctors from other cities and prefectures to avoid movement of patients. 15) Our COVID-19 patients were insured by our structures with the creation of a dialysis unit dedicated to COVID-19 positive. 16) Chemoprophylaxis with Chloroquine was instituted when contact with a COVID-19 patient was suspected.

Sir, we bring you our experience based on our means and the infrastructure available and based on national recommandations of the Moroccan society of nephrology, which remains modifiable with the evolution of the disease and which will undoubtedly impose other rules to be implemented in POST COVID-19 [2,3]. Since the first case declared in Morocco on March 1st and until the date of submission of the work with the containment measures maintained for 3 months we don´t declare in our center any cases of COVID-19 positive patients or cases of staff affected either.

## Competing interests

The authors declare no competing interests.
